# Lean oncology: a new model for oncologists

**DOI:** 10.1186/1479-5876-10-74

**Published:** 2012-04-25

**Authors:** Vincenzo Montesarchio, Antonio Maria Grimaldi, Bernard A Fox, Antonio Rea, Francesco M Marincola, Paolo A Ascierto

**Affiliations:** 1 Unit of Medical Oncology, Ospedale "D. Cotugno" A.O.R.N. dei Colli, Via G. Quagliariello, 54, 80131, Naples, Italy; 2 Unit of Medical Oncology and Innovative Therapy, Istituto Nazionale per lo Studio e la Cura dei Tumori "Fondazione G. Pascale", Via Mariano Semmola, 80131, Naples, Italy; 3 Earle A Chiles Research Institute, Robert W Franz Research Center, Providence Portland Medical Center, and Department of Molecular Microbiology and Immunology, Oregon Health and Science University, Portland, OR, USA; 4 Infectious Disease and Immunogenetics Section (IDIS), Clinical Center and trans-NIH, Bethesda, MD, USA

## Abstract

The history of the term Lean is relatively recent and originates from the Toyota Production System (TPS). The term "Lean" means "thin", which refers to a mental process, operational, productive, no-frills, quick but not hasty, consequential to the previous event. The Lean process flows seamlessly into the result, eliminates unnecessary complications to the effect, prevents unnecessary equipment processes. The idea is to 'do more with less', like using the (few) available resources in the most productive way possible, through the elimination of all types of waste that inevitably accompanies every stage of a production process. Lean management is primarily a management philosophy, a system of values and behaviors that goes beyond the mere application of the instrument and that, once internalized, will form the nucleus of the corporate culture. "Lean Oncology" is a term coined to identify a methodology of care and treatment to cancer patients, consisting on process simplification, streamlining of the organizational and routes of drug treatment, detection and elimination of waste. Its main objective is the centrality of the patient.

## Why "lean" in oncology?

The term Lean is a synonym of simplicity, as well as efficiency. The same Leonardo Da Vinci believed that Simplicity is the highest refinement, and Albert Einstein stated that it is essential to make things as simple as possible, but not the easy way.

Equity is a derivation of the principles of social justice and the State has the duty to ensure the satisfaction of basic needs to everyone. The task of the community in pursuing fairness can be understood in terms of egalitarianism, that is intended as the duty of the State to guarantee everyone the same opportunities in terms of health care, in obtaining the minimum standard of health services.

International development of health technologies keeps pace with the continuous transformation of society in terms of appearance of new diseases, increase of patient education and aging population. This process requires an evolution of care based on appropriateness and fairness of the offer. The expansion of health care spending is a widespread phenomenon in all the institutional and organizational contexts that characterize the modern health systems. The health services must take into account limited resources to avoid that health will no longer be an asset available to all due to cost explosion.

One of the emerging issues in oncology, related to the wave of optimism that comes from improvements in several types of cancer from the high cost of new anticancer drugs, is the overall assessment of the innovative drug therapies in oncology and, in particular, their clinical, economic and ethical, to reconcile on the one hand the guarantee of a fair and uniform patient access to such care, and secondly the need to allocate resources according to appropriateness criteria, perhaps by providing forms of co-responsibility with the patient and his family, and always taking into account the issue of economic viability and compatibility of evidence-based ethical choices, even in reference to patients with other diseases.

But the other problem to be solved concernes waste. Nowadays, nobody makes the necessary distinction between cost and waste. Medicine is responsible for setting and achieving its objectives (effectiveness), economics and health policy to apply its principles (efficiency). The cooperation of the two sciences is necessary in order to maximize the collective welfare. Economic efficiency concerning the combination of factors indicates that best result (output) can be obtained from the resources (inputs) available, and refers to the incentives to minimize costs by individual production units.

Looking to waste, this is easily identifiable but hard to eliminate (due to habits, organizational powers, etc.). It is possible to identify different types of waste such as pharmacological, organizational and procedural. The fundamental rule in the management of waste is: "The reduction of waste increases the capital". Waste can be further distinguished according to the type of waste in: Defects (lack of quality), Excessive production (greater than what required at the time), Transport (unnecessary travel), Waits (material still waiting to be processed), Stocks (in general the stock is always a waste), Unnecessary operations (which is not value added), the process itself. Typical examples of waste in Oncology are adverse reactions to inappropriate premedications, unnecessary PET and CT, improperly used high-cost drugs, excessive use of growth factors, inappropriate use of antiemetic therapy, or "maintenance" without scientific evidence. Some of these are children of the so-called defensive medicine such as the duplication or redundancy of diagnostic tests not really useful and effective for a certain disease, or the Therapeutic Obstinate Chemotherapy in the terminal phase just to satisfy the hopes of the family, when they prefer hearing: "We can do something," rather than saying There is nothing we can do."Lean Oncology" is a term coined to identify a methodology of care and treat cancer patients, consisting on process simplification, streamlining of the organizational and routes of drug treatment, detection and elimination of waste. Its aim is the wellbeing of the patient, who is the centre of this process. The history of the term Lean is relatively recent and originates from the Toyota Production System (TPS). This is the result of a production philosophy that is closely affected by cultural influences of the environment in which it arises: Japan. The discipline, the continuous search for perfection, the care for detail and research are the most effective background of the culture of the samurai. In the mid-twentieth century, President and CEO of Toyota Motor Company introduced innovative principles in the field of production that go under the name of Lean Manufacturing or Lean Production, a term coined in 1988. The first time managers around the world heard of the revolutionary TPS was at the end of 1990, with the book "The Machine That Changed the World. The idea is to 'do more with less', i.e. to use the (few) available resources in the most productive way possible through the elimination of all types of waste (Muda) that inevitably accompanies every stage of a production process. The term "Lean" means "thin",which refers to a mental process, operational, productive, no-frills, quick but not hasty, consequential to the previous event. The Lean process flows seamlessly into the result, eliminates unnecessary complications to the effect, prevents unnecessary equipment processes. The primary objective of the Lean management is the maximization of customer value and the reduction of resources required to generate it. In order to achieve this objective, it is necessary to restructure the whole process according to 5 basic principles (value, value stream, continuous flow, pull, perfection) and then, through the removal of waste and the spread of the culture of continuous improvement and problem solving, free the best business resources for reuse in new strategic challenges, ensuring mutual growth. The term "Lean" can be applied to any aspect of life (Lean Thinking, Lean Production, Lean Office, Lean Management, Lean Healthcare, etc.). The fundamental principle of Lean Production is the hunting of waste: anything not used to increase the value of the product as perceived by the customer and that the customer is willing to pay is considered waste and, as such, must be eliminated. In order to build a lean production it is necessary to identify the best value stream that leads to the result, team play action for more actors, standardize routes, improve standardization and reduce errors. Lean is quick and simple; if you manage a process with Lean Thinking, you dont need to have lots of space to make a smooth production process, but it is sufficient that every actor knows the times and manner of its action (OSS, Nurses and Doctors).

The world itself is in "Lean" or looks for more and more regulatory simplification. In Italy the majority of industrial companies (including health) is currently experiencing a situation of inadequacy of logic with respect to operational and organizational dynamics of the market.

The health care companies have realized that the most careful adjustment goes through a deep revolution of its structure-oriented simplification and speeding up of operational and management processes. The ideas of Lean Healthcare and Lean Oncology provide organizational simplification of access to care, slimming treatments, waste reduction (avoid non-useful therapies - EBM, high-cost therapies grouped in the same day), appropriateness of prescribing medication support, computerization and reduction of printed media, reduction of errors, appropriateness of admissions, health technology assessment, appropriate use of Target Therapy. The cost-cutting "tout court" inevitably leads to a reduction of the output in terms of quality of healthcare received by the patient. For the Lean Revolution theres the need of a change in management thinking, based on the collaboration between economists and health professionals, with an absolute elimination of the personalisms, so that the patient is the center of the service offered.

## Be lean or do lean?

You cannot just "do" Lean: Lean transformation encompasses all operational areas of a reality and cannot be seen as a "project" or a program to reduce costs in the short term. In this sense, the Lean management is primarily a management philosophy, a system of values and behaviors that go beyond the mere application of the instrument and that, once internalized, will form the nucleus of the corporate culture.

